# Acute diarrhoea due to a Shiga toxin 2e-producing *Escherichia coli* O8 : H19

**DOI:** 10.1099/jmmcr.0.005099

**Published:** 2017-06-14

**Authors:** Angela Saupe, Birgit Edel, Wolfgang Pfister, Bettina Löffler, Ralf Ehricht, Jürgen Rödel

**Affiliations:** ^1^​ Institute of Medical Microbiology, Jena University Hospital, Jena, Germany; ^2^​ Alere Technologies GmbH, Jena, Germany

**Keywords:** STEC infection, diarrhoea, antibiotics, azithromycin

## Abstract

**Introduction.** Identification of non-O157 Shiga-toxin-producing *Escherichia coli* (STEC) infections may be underestimated in microbiological diagnosis.

**Case presentation.** A 58-year-old woman developed diarrhoea with watery and subsequently mucous stool. Initial multiplex PCR testing revealed a positive result for *stx_2_*. Culture isolation of a STEC was successful only after repeated inoculation of chromogenic *E. coli* media. Molecular characterization was performed and identified the isolate as *stx*_2e_-positive STEC of serotype O8 : H19. The strain harboured *lpfA*, but not *eae*.

**Conclusion.** This case highlights the usefulness of initial multiplex PCR for diagnosis of non-O157 STEC infection.

## Abbreviations

EHEC, enterohaemorrhagic Escherichia coli; LP, long polar; STEC, Shiga-toxin-producing Escherichia coli.

## Introduction

Shiga-toxin-producing *Escherichia coli* (STEC) are important food-borne enteric pathogens. There are several Shiga-toxin (Stx) variants that have been associated with various clinical manifestations; in particular, strains with the combined presence of *stx*_2_ and *eae* (intimin) genes are often responsible for severe outcomes, such as haemorrhagic colitis and haemolytic uraemic syndrome [1]. The major pathogen of this group belongs to the serotype O157. There is a growing number of non-O157 STEC in humans that have been isolated from patients in clinical cases and from outbreaks [2–5]. Because non-O157 STEC can possess uncommon serotypes and are often not distinguishable from non-pathogenic *E. coli* on selective and chromogenic media, they are difficult to isolate and remain under-recognized in the laboratory. In this report, we present a symptomatic case caused by a *stx*_2e_-expressing STEC of serotype O8 : H19 that would not have been diagnosed without pre-screening of the stool sample with multiplex PCR.

## Case report

In early summer of 2016, a 58-year-old woman experienced watery diarrhoea and vomiting. The patient had spent a weekend with her family in the north of Germany. Two days before disease onset, she had eaten poultry liver for lunch and grilled swine meat at an evening barbecue. Other family members also consumed the swine meat, but did not develop any symptoms. The patient's medical history was significant for diabetes mellitus, but no other risk factors. A mild diet led to recovery after 2 days. After shifting to a normal diet, she felt sick again with developing watery diarrhoea. Replacement of diet and symptomatic treatment did not lead to any improvement, and during the next few days she produced mucous stool and suffered from abdominal pain, tenesmus and fatigue.

After 1 week of disease, the patient visited a medical practitioner and a stool sample was analysed in the laboratory. An initial rapid test using the Verigene EP assay (Luminex, purchased from Thermo Fisher) showed a positive result for *stx_2_*. The test was negative for *stx_1_*, *Salmonella* spp., *Shigella* spp., *Campylobacter* spp., *Yersinia enterocolitica*, *Vibrio* spp., norovirus and rotavirus.*Clostridiumdifficile* tested negative by single PCR (BD Max; BD). For culture, the stool sample was streaked onto *Salmonella–* *Shigella*-agar, Hektoen agar, Butzler agar, CIN agar and Brilliance *E. coli*/coliform chromogenic agar (all plates were purchased from Oxoid, Thermo Fisher Scientific). For enrichment of pathogenic bacteria, Gram-negative (GN) broth and selenite broth (BD) were inoculated. On one hand, culture confirmed the negative results of the Verigene assay; on the other hand, *E. coli* colonies isolated on chromogenic agar did not agglutinate with polyspecific antibody reagents directed against frequent serotypes (Sifin Diagnostics) and tested negative for *stx*_2_ using the RIDAGENE EHEC/EPEC real time multiplex PCR (R-Biopharm). Agglutination and PCR were also performed from the inoculum site using a mixture of colonies. Brilliance *E. coli*/coliform agar was inoculated again with stool and GN broth samples, and on the following day testing of colonies for *stx*_2_ by PCR was positive for the original stool specimen and GN broth. *stx*_1_ and *eae* genes were not detected. Agglutination with polyclonal antisera covering 22 O serotypes (O25, O26, O44, O55, O78, O86, O91, O103, O111, O114, O118, O119, O124, O125, O126, O127, O128, O142, O145, O157, O158 and O164) also remained negative. Species identification was performed by MALDI-TOF MS (Vitek-MS) and biochemistry (Vitek-2). The strain was positive for β-galactosidase, β-glucuronidase and sorbitol fermentation. The patient was treated with azithromycin (500 mg day^−1^) for 3 days and recovered after 2 days without any relapse.

## Investigations

To identify the unusual serotype and additional virulence factors, the STEC isolate was characterized using the *E. coli* PanType AS2 genotyping kit (Alere Technologies). This hybridization array includes several hundred sets of oligonucleotides for DNA-based detection of virulence and resistance genes, and it covers genes for the identification of 23 epidemiologically relevant O-antigens, as well as 47 H-antigens. The Shiga toxin was identified as *stx*_2e_, which is rarely observed among human isolates [2, 6]. Serotypes O8 and H19 were identified. The strain was negative for *eae* and type III secretion system (T3SS) genes, but positive for *lpfA*, encoding long-polar (LP) fimbriae that are associated with diarrhoeagenic strains of *E. coli* [7, 8]. Three resistance genes were detected: *strB*, encoding aminoglycoside-6′′-phosphotransferase; *tetA*, encoding tetracycline efflux protein; and *bla*_TEM_, encoding a β-lactamase. Phenotypic resistance was found against ampicillin and piperacillin.

## Discussion

This case describes the rare isolation of a *stx*_2e_-producing *E. coli* from a patient with acute diarrhoea and vomiting. Only about 1 % of STEC infections in humans have been attributed to *stx*_2e_ [9]. Pigs are known to carry *stx*_2e_-positive *E. coli* causing oedema disease. The isolated *E. coli* strain belonged to a group of *eae-*negative STEC that express *stx*_2_ and alternative adhesins such as LP fimbriae. LP fimbriae have been described in STEC/enterohaemorrhagic *E. coli* (EHEC) and enteropathogenic *E. coli* (EPEC), and they are also present in O157 : H7 strains. The isolate was of serotype O8 : H19. Unusual serotypes may not be covered by the commercial polyclonal antisera used for standard diagnosis of human infections. Therefore, screening of colonies in cultures should be performed by *stx* PCR. Serotype O8 : H19 is characteristic for porcine *stx*_2e_-producing *E. coli*, so pork meat may have been the source of infection in the present case [6, 10]. The patient was treated with azithromycin because of recurrence of diarrhoea and impairment after stopping a mild diet. Azithromycin was successfully applied in patients with EHEC infections during the German O104 EHEC outbreak and has been shown to reduce the time of shedding bacteria [11]. The patient benefited from the azithromycin therapy rapidly.

This case of STEC infection would not have been diagnosed without an initial culture-independent PCR multiplex assay. Low levels of STEC in stool samples may be responsible for the failure to isolate such strains in culture. The STEC was isolated only after repeated inoculation of *E. coli*/coliform chromogenic media. Because the strain was positive for β-galactosidase, β-glucuronidase and sorbitol fermentation, its colonies could not be distinguished morphologically from *E. coli* background flora. Therefore, this case highlights the usefulness of a culture-independent rapid molecular test that provides a diagnostic result without significant time delay. The Verigene EP microarray test used in our laboratory is an U.S. Food and Drug Administration (FDA)-cleared microarray test with automated processing. The test running time is about 2.5 h, with less than 5 min of hands-on time, including sample preparation. The implementation of such tests for routine stool diagnostics depends on the costs for machines and consumables. These factors may limit the general use for all samples, but the implementation for cases with the need of sensitive and rapid diagnosis is an important option for the clinical microbiology laboratory [12, 13]. In 2016, we performed 34 tests in cases of acute diarrhoea with 12 positive results. In four cases, no isolate could be obtained from culture, including for one case of STEC (Table 1). Multiplex PCR assays for diarrhoeagenic bacteria may not only be used as rapid screening tools but also be reasonable tests in cases with negative culture and virus PCR despite acute diarrhoea.

**Table 1.** Comparison of Verigene EP and culture results

	Culture positive	Culture negative
Verigene EP positive	8*	4†
Verigene EP negative	0	22

* *Salmonella* spp. (5), *Campylobacter* spp. (2), STEC (1).

† *Salmonella* spp. (1), *Shigella* spp. (1), *Campylobacter* spp. (1), STEC (1).
